# Targeted Metabolomics: The LC-MS/MS Based Quantification of the Metabolites Involved in the Methylation Biochemical Pathways

**DOI:** 10.3390/metabo11070416

**Published:** 2021-06-24

**Authors:** Georgia Ntasi, Anthony Tsarbopoulos, Emmanuel Mikros, Evagelos Gikas

**Affiliations:** 1 Division of Pharmaceutical Chemistry, Faculty of Pharmacy, National and Kapodistrian University of Athens, Panepistimiopolis Zografou, 15771 Athens, Greece or georgiant91@gmail.com (G.N.); mikros@pharm.uoa.gr (E.M.); 2 Department of Pharmacology, Medical School, National and Kapodistrian University of Athens, Mikras Asias 75, 11527 Athens, Greece; atsarbop@med.uoa.gr; 3 The Goulandris Natural History Museum, Bioanalytical Laboratory, GAIA Research Center, 13 Levidou Street, 14562 Kifissia, Greece; 4 Laboratory of Analytical Chemistry, School of Chemistry, National and Kapodistrian University of Athens, Panepistiomiopolis, Zografou, 15771 Athens, Greece

**Keywords:** methylation, Liquid Chromatography-Tandem Mass Spectrometry (LC-MS/MS), solid phase extraction, validation

## Abstract

Biochemical methylation reactions mediate the transfer of the methyl group regulating vital biochemical reactions implicated in various diseases as well as the methylation of DNA regulating the replication processes occurring in living organisms. As a finite number of methyl carriers are involved in the methyl transfer, their quantification could aid towards the assessment of an organism’s methylation potential. An Hydrophilic Interaction Chromatography-Liquid Chromatography Multiple Reaction Monitoring (HILIC-LC-MRM) mass spectrometry (MS) methodology was developed and validated according to Food & Drug Administration (FDA), European Medicines Agency (EMA), and International Council for Harmonisation of Technical Requirements for Pharmaceuticals for Human Use (ICH) for the simultaneous determination of nine metabolites i.e., B12, folic acid, 5-methyltetrahydrofolate, S-adenosylmethionine, S-adenosylhomocysteine, betaine, phosphocholine, N,N-dimethylglycine, and deoxythymidine monophosphate in human blood plasma. The sample pretreatment was based on a single step Solid-phase extraction (SPE) methodology using C18 cartridges. The methodology was found to accurately quantitate the analytes under investigation according to the corresponding dynamic range proposed in the literature for each analyte. The applicability of the method was assessed using blood donor samples and its applicability demonstrated by the assessment of their basal levels, which were shown to agree with the established basal levels. The methodology can be used for diagnostic purposes as well as for epigenetic screening.

## 1. Introduction

Biochemical methylation reactions [1] (although the non-trivial names methylation cycle [2] and transmethylation are sparsely used), comprise the swarm of biochemical pathways that manage methylation signaling molecules, contributing to a wide range of bodily critical functions through the controlled transmission of methyl groups to amino acids, proteins, enzymes, and DNA. 

The methylation cycle can be thought of as consisting of five major biochemical pathways, their interconnection being summarized as follows: (a) the tetrahydrobiopterin cycle which involves the conversion of 7,8-dihydrobiopterin (BH2) to 5,6,7,8-tetrahydrobiopterin (BH4) with the transport of the methyl group (Me) to 5-Me-tetrahydrofolate (5-Me-THF) through the action of methylenetetrahydrofolate reductase (involving the synthesis of tryptophan from serotonin and tyrosine from dopamine) [3]; (b) the folate cycle—where the Me is transferred to folate with the formation of 5-Me-tetrahydrofolate, mediated by B12 [4]; (c) the methionine cycle with the cycling of Me from methionine to S- adenosyl-methionine (SAM) concomitant with S-adenosyl-homocysteine (SAH) to homocysteine by accepting Me through B12 [5]; (d) the trans-sulfuration cycle with the disposition of Me to cystathionine towards cysteine formation [6]; and finally (e) the urea cycle as arginine is converted to citrulline through the action of BH4 on endothelial nitric oxide synthase eNOS [7]. 

Methylation is central to the biochemical regulation of folic acid (B9) [8,9,10] and vitamin B12 [11,12] levels, which are responsible for the synthesis of nucleic acids and neurotransmitters, therefore any dysregulation causes a variety of nervous system diseases [13]. 5-Methyltetrahydrofolate has been used as a complementary treatment in the case of selective serotonin reuptake inhibitor (SSRI) treatment of severe depression [14,15] with obesity being a co-morbidity factor [16,17]. Elevated urinary levels of betaine correlate with increased risk of cardiovascular disease [18], whereas both betaine and dimethylglycine are positively correlated with the annihilation of pentylenoterazole seizures in experimental animals [19]. Phosphocholine undergoes upregulation in human breast cancer incidents [20], whereas IgA antibodies against phosphocholine have been proven to associate with the long-term risk against cardiovascular disease [21]. *S*-adenosyl-l-methionine (SAM), is used as a prescription drug in some countries as it has chemoprotective effects against liver disease [22]—albeit controversial due to lack of large-scale evidence—whereas it exhibits limited antidepressant efficacy [23]. SAH shows clinical importance as a cardiovascular disease biomarker [24], also being involved in the establishment of neurodegenerative diseases such as Alzheimer’s [25].

Nevertheless, the main biological regulation pathway of the aforementioned metabolites is their participation in the regulation of the DNA replication through Cytosine (C) and histone methylation, leading to their local untwist thus inducing DNA replication. Therefore, methylation reactions are central to epigenetics [26,27]. DNA methylation is exceedingly complex but the enrichment of the results stemming from the recent advances in methylation analysis technology at the metabolic level, could well enhance our understanding of this process. 

Many methodologies dealing with the quantification of the aforementioned substances appear in the literature, nevertheless their simultaneous determination still remains a demanding task, largely due to their chemical diversity and the significant differences in their levels of plasma. These methodologies have so far deployed multiple separate chromatographic, and/or detection schemes for the quantification of metabolite intermediates of one or maximum two methylation dependent cycles. In particular, most of the studies involving methylation related metabolites have been based on the detection of folate and/or methionine metabolite intermediates [27,28,29,30,31,32,33,34,35]. Furthermore, other approaches include the study of trans-sulfuration and/or urea cycle metabolite intermediates [35,36,37,38] or vitamins [38,39,40]. However, no single analytical method currently exists to monitor both metabolites and co-factors spanning all the five methylation transport pathways (tetrahydrobiopterin, folate, methionine, trans-sulfuration, urea cycle). To address this limitation, we have developed a new methodology for the quantification of eight key methylation metabolites. In particular, a UHPLC-MS/MS methodology was developed for the simultaneous quantification of B12, B9, 5-methyltetrahydrofolate, SAM, SAH, betaine, phosphocholine, N,N-dimethylglycine (DΜG), and the deoxythymidine monophosphate (dTMP).

Development of an analytical methodology for the quantification of metabolites associated with different pathways is a challenging task. The large variance in the basal levels of these metabolites in plasma requires a fairly wide calibration range in order to detect and quantify adequately the metabolites thereof. For instance, the basal level of B12 demands that the lower limit of the linear model should be equal or below 0.1 ng/mL for a reliable detection and should reach over 3000 ng/mL due to the basal levels of betaine. Furthermore, some metabolites such as the folate cycle intermediates exhibit high instability that leads to chemical degradation under various conditions of temperature, pH, oxygen, or light [40,41,42,43]. For this reason, their analysis is usually performed under alkaline conditions where for instance SAM and SAH are not stable (optimum pH 4–5) although betaine in acidic conditions has two forms, one positively charged and one double-charged [43,44]. Thereof, the incorporation of all molecules in a single method is quite tricky and challenging. 

The motivation for this project stems from the concept to study biochemical metabolite pathways, as integrated entities, in a targeted fashion affording a holistic view. The untargeted metabolomic approaches often lead to severe difficulties in assigning structures to the putative metabolites hindering their usefulness, whereas the extensive targeted approaches, exhibit practical difficulties in the acquisition of standard compounds both due to their cost and availability. Furthermore, the latter methodologies usually suffer from extreme complexity as well as from a variety of inherent issues of complex methodologies such as unpredictable matrix effect or compromised specificity. The current methodology represents a new notion in metabolomics, i.e., a targeted metabolomic focus based on biological pathways rather than chemical classes. As methylation represents a major pathway for health and disease, as well as for the exploration of fundamental biological phenomena and the interaction of the organisms with the environment, it is challenging to explore this idea as proof of concept.

## 2. Results

### 2.1. Validation Results

The validation results were in accordance with the acceptance/exclusion criteria set. The limitations regarding stability and robustness could be addressed by the careful control of the procedure. 

#### 2.1.1. Calibration Model

The calibration models for all analytes exhibited adequate fit to the experimental data, all the regression coefficients being >0.99, whereas the BCV’s (Back Calculated Values) were well within the acceptance criteria (Table 1).

Nevertheless, as B12 followed an acceptable calibration model only at high concentrations (>5 μg/mL), the current methodology is not capable of measuring it in biologically meaningful concentrations (0.2 ng/mL) [28] and was excluded. Presumably, as B12 is a large lipophilic molecule, it is not adequately eluted under the SPE conditions applied.

#### 2.1.2. Recovery

The recovery values ranged between 47 and 102%, with the differences being attributed to the vastly different chemical structures of the analytes. Nevertheless, the corresponding %RSD values show adequate reproducibility (<15%), rendering the method acceptable (Table S1).

#### 2.1.3. Specificity

The specificity study exhibited that the plasma samples obviously showed endogenous levels of the analytes, therefore no blank chromatograms were found. On examining for possible interferences, the standard solutions, as well as two eluates from the SPE procedure employing only standards, no signal >20% of the LOD at the respective retention time was found, hence the signal observed resulted from the endogenous metabolite levels.

#### 2.1.4. Accuracy

The results showed that the methodology fulfilled all the acceptance criteria (Table S2).

#### 2.1.5. Precision

The precision was calculated as repeatability and intermediate precision and found to be compliant with the acceptance criteria (Table S3).

#### 2.1.6. Sensitivity

The sensitivity assessed by the proposed methodology as defined by the LLOQ (Lower Limit of Quantitation) was 80 ng/mL for 5-MeTHF, 15 ng/mL for folic acid, SAM, SAH, and d-TMP, 800 ng/mL for phosphocholine, 150 ng/mL for DMG, and 3000 ng/mL for betaine. The corresponding instrumental values were 25 ng/mL for 5-MeTHF, 5 ng/mL for folic acid, SAM, and SAH, 10 ng/mL for d-TMP, 250 ng/mL for phosphocholine, 50 ng/mL for DMG, and 5 ng/mL for betaine (Table S4). 

#### 2.1.7. Matrix Effect

The matrix effect tabulated in Table S5 was found to be acceptable, as the LLOQ levels achieved for each analyte were adequate. 

#### 2.1.8. Stability

The calculated %RSD values for the freeze-thaw stability (Table S6a) were >15% indicating that the samples should be analyzed immediately after thawing, whereas the autosampler stability indicated that the substances are stable when they remain processed under 8 °C (Table S6b).

Overall, the unprocessed samples should be refrigerated, but after the SPE processing they are stable enough to be analyzed in large batches, indicating that the bottleneck of the analysis is the capability to perform the SPE in due time.

#### 2.1.9. Dilution Integrity

The %Er for all analytes was <15%, indicating that the methodology can be used for samples with levels exceeding a 10-fold level higher than the ULOQ (Upper Limit of Quantitation) of each analyte.

#### 2.1.10. Incurred Samples Reanalysis

All samples were found to comply with the specifications of the regulatory body fulfillments.

#### 2.1.11. Robustness

The data indicate that the variables examined do not exceed the exclusion criteria (<15%) except the buffer concentration, probably due to the pH-depended zwitterionic nature of the analytes. Therefore, careful control of the buffer preparation is recommended (Table S7). 

### 2.2. Application to Human Plasma Samples

The developed methodology was applied to plasma samples from anonymous blood donators. The method should be able to assess the analyte basal levels, as the exclusion criteria for the donors deter individuals with known illnesses from participating in the process. The basal levels were determined by extrapolation of the calibration curves to the x axis and the point of intercept was considered as the endogenous level of the metabolite. The calibration model was approximated, when necessary, by the linear part, determined by those points showing a correlation coefficient >0.99 (Table S8).

Using the methodology described the levels of each metabolite in the analyzed plasma can be found in Table 2.

## 3. Discussion 

Through a thorough bibliographical search, it is evident that the methodologies described in the literature use blank plasma. This is in our opinion contradictory as there is no chance of discovering individuals without endogenous levels of these metabolites. Therefore, the validation processes described in the literature should be re-evaluated taken also into account that no metabolite striping methodology (i.e., plasma steroid depletion by activated charcoal as in the case of endogenous steroids) has so far been described. The standard addition methodology used in our study does not require blank samples whereas it uses a pooled sample from six healthy plasma sources. Therefore, all analytes correspond to the population mean. As a result, the method described herein does not rely on the creation of blank samples, which is not feasible and therefore could be characterized as reliable. 

To explore the pH dependence of the zwitterionic analytes, the computational approach (https://chemicalize.com/welcome, access on 15 July 2016), showed that when the pH reaches below 2.5, all analytes are positively charged, securing ionic chromatographic interactions. On employing this approach all substances were adequately chromatographed (tailing factor < 1.3) on an HILIC column, whereas the fused core 2.7 μm technology (vs. 3 μm c-ZIC-HILIC) enhanced the chromatographic resolution.

**Figure 1:**
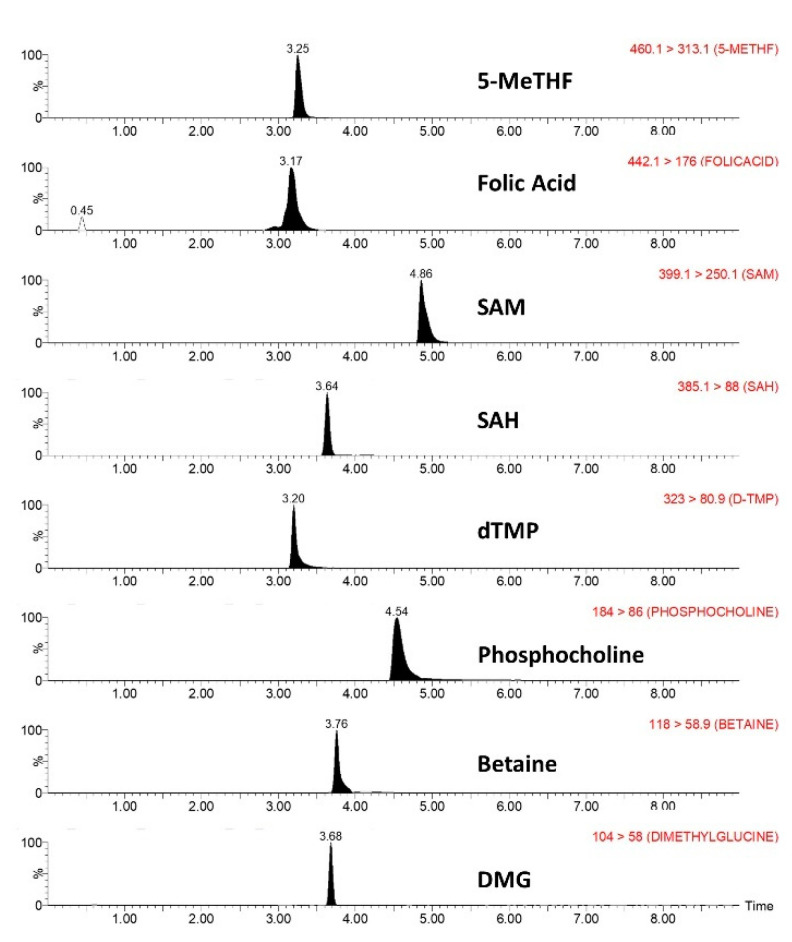
Representative UPLC–ESI MS/MS chromatograms of plasma samples spiked with the analytes.

Various sample preparation methodologies were evaluated (protein precipitation, LLE and various SPE packing materials). As the analytes were positively charged at pH 5, the sample loading aliquot from a C18 SPE cartridge (pH 5 using ammonium acetate) was collected for the LC-MS/MS analysis. The analytes, being positively charged, are directly eluted from the cartridge as opposed to the medium and high lipophilicity plasma substances, which are retained by the highly hydrophobic packaging material. Adopting this “indirect” cleaning procedure all the analytes were recovered exhibiting a high ratio, at the expense of B12 which was strongly retained due to its lipophilicity [45]. The analytes were separated by the developed UPLC separation methodology with a resolution R > 1.5, in order to minimize cross-contribution to the signal and any incidental matrix effect. The efficient separation among the analytes is shown in the representative UPLC–ESI MS/MS chromatograms of plasma samples spiked with the analytes (Figure 1). 

The data analysis procedure was conducted using QuanLynx (Waters, Milford, CT, USA) and flagging invoked in mock samples (samples spiked with selected analytes in various levels) worked adequately for all analytes. Data analysis proceeded seamlessly for all samples, indicating that the procedure can be readily integrated into clinical analysis. Nevertheless, the methodology should be applied in much larger cohorts to acquire further information about its applicability domain. 

The results obtained from blood donor samples are consistent with the values determined for the general population indicating that the methodology is applicable in the normal population domain. The applicability area applies to two major areas: the assessment metabolite deficiencies for diagnostic reasons (the current methodology enables an integrated view of biochemically related metabolites e.g., the SAM–SAH interplay but also interrelates pathways not related so far) and (in our opinion more interestingly) the monitoring of the methyl group transport assessing the levels of the participating metabolites. Therefore, up/down-regulation of some or all the metabolites in the methyl transportation pathway, potentially means up- or down-regulation of the cellular methylation potential, e.g., some kind of upregulation could potentially mean the initialization of carcinogenesis at the cellular, thus at the subclinical, level. Therefore, the methodology could potentially serve as a biomarker panel for early-stage diagnosis of diseases related to cancer. Finally, the methodology can be considered as a third mode of metabolomic analysis, we call focused metabolomics, as it is markedly different from untargeted (aiming to analyze as many analytes are possible—only the metabolite level ratio can be assessed in comparison among the experimental groups) but also targeted metabolomics (full validation according to the regulatory body protocols of selected metabolites—currently not feasible for hundreds of metabolites). Therefore, the focused metabolomics are targeting one or a series of biochemical pathways instead of a chemical class. Therefore, the current methodology could aid in the augmentation and verification of the genomic methodologies for assessing the epigenetic potential of a target organism.

## 4. Materials and Methods

### 4.1. Chemicals and Reagents

Reference analytical standards for S-adenosylmethionine, S-adenosylhomocysteine, betaine, phosphocholine, N,N-dimethylglycine, folic acid, 5-methyltetrahydrofolic acid, B12 (cyanocobalamin), dTMP and daptomycin used as the internal standard (IS) were purchased from Sigma Aldrich. Ammonium formate, formic acid, and methanol were of LC-MS grade (Sigma Aldrich, St. Louis, MO, USA), whereas LC-MS grade acetonitrile was obtained from Carlo Erba (CARLO ERBA Reagents S.A.S., Val de Reuil, France). Citric acid and 1,4-dithioerythritol (analytical grade purity) were purchased from Sigma. HyperSep C18 cartridges (50 mg–1 mL) were obtained from Thermo-Fischer Scientific (Waltham, MA, USA).

Stock standard solutions for SAM, SAH, betaine, phosphocholine, N,N-dimethylglycine, B9, 5-methyltetrahydrofolic acid, B12, and dTMP were prepared at the 1 mg/mL level, whereas that of IS at a concentration of 500 μg/mL and diluted in 50% methanol/water (vol/vol) with 10% citric acid and 1,4-dithioerythritol due to long-time stability issues. Combined aliquots of the stock standard solutions were prepared at four concentration levels, i.e., 25, 250, 2500, and 25,000 ng/mL at the beginning of each laboratory week and kept refrigerated at −30 °C. At least three QC levels namely QCL (Low Quality Control), QCM (Medium Quality Control), QCH (High Quality Control) along with the respective LLOQ (Lower Limit Of Quantification) and ULOQ (Upper Limit Of Quantification) were used for each analyte. Because the calibration range covered the area 2.5–50,000 ng/mL some of the analytes encompassed more than the aforementioned three QC levels for the validation purposes. The internal standard used was daptomycin in methanol (final concentration added to the samples 10 μg/mL). The QC levels employed for each analyte can be found in the Supplementary Tables. All calibrators and QCs were prepared in plasma. 

### 4.2. Plasma Sample Collection and Pretreatment

Human plasma samples were generously donated from the National Center for Blood Donation of Greece (EKEA) from anonymous healthy donors registered to the Greek National Blood Donor Registry and were stored at −80 °C until analysis. Due to the low levels of many analytes in the blood plasma a solid phase extraction procedure was employed. Thus, 50 μL of thawed plasma, 100 μL of acetonitrile, 200 μL of 5 mM ammonium formate buffer pH 5, 10 μL of internal standard, and 10 μL of the appropriate working standard solution were mixed, vortexed thoroughly, and applied to the solid phase extraction cartridge. Before the sample application each cartridge was washed with 1 mL of acetonitrile and equilibrated with 1 mL of 5 mM ammonium formate buffer adjusted to pH 5. The eluate was collected and 10 μL was analyzed. 

### 4.3. Chromatography

An Acquity^®^ UPLC system (binary solvent and solvent manager, column oven) (Waters) was employed for the chromatographic separation on a Speed Core Hilic (2.1 × 100 mm, 2.6 μm) column (Fortis). The gradient elution methodology was utilized with mobile phase (A) being aq. ammonium formate (5 mM—pH 5) and (B) acetonitrile.

The elution program was started with 99.9% (B) for 2 min, increased to 40% and held for 2 min, reached 6% B for an additional 2.30 min, and then returned to 99.9% to re-equilibrate. The total injection-to-injection run time was 9 min. The flow rate was 0.7 mL/min and the column oven temperature was set at 40 °C, maintained throughout all experiments.

### 4.4. Mass Spectrometry

A Waters Acquity TQD^®^ tandem mass spectrometer (Waters; Waters Corporation, Milford, MA, USA) was used for mass spectrometric analysis. MassLynx NT version 4.1 was used for system control, whereas the application manager TargetLynx was used for data processing. The mass spectrometer operated in the positive electrospray ionization mode with the following parameters: capillary voltage 3.5 kV, source temperature 150 °C, desolvation temperature 600 °C, gas flow 650 L/h. The transitions (multiple reaction monitoring—MRM—mode), dwell time, collision energies, and capillary voltages employed are shown in Table 1. The mass spectrometric parameters were determined by infusion at the 50 μg/mL level (flow rate of 15 μL/min) and the Intelistart embedded software was used to record cone voltage and collision energies of the highest two MS/MS transitions discovered (Table 3).

The analytes were separated with a resolution R > 1.5, in order to minimize cross-contribution to the signal and any incidental matrix effect between them (Figure 1). 

### 4.5. Validation 

Due to the lack of blank plasma samples and the varying basal levels, 20 batches of plasma (healthy blood donators) were acquired and homogenized. The pooled plasma depot was used throughout the validation as representative of the plasma from the general population. These samples are called pooled samples. The standard addition methodology was employed for constructing the spiked plasma levels. The underlying idea is to develop a sensitive enough method for calculating the basal levels of the healthy population, therefore having the ability of differentiating any up- or down-regulated levels of the analytes. It should be noted that the literature search did not reveal any substance depletion methodology and surprisingly, despite the fact that there is no blood plasma devoid from these substances, this did not seem to have been considered in the so far developed methodologies. The method was validated according to FDA, EMA, and ICH. The response used was the ratio of the area of the chromatographic peak of each analyte to that of the internal standard. 

#### 4.5.1. Specificity

As the plasma samples are not devoid of analyte endogenous levels, a signal is expected. In order to exclude any interference from the solvents etc. employed, the interferences were assessed for the standards, whereas the peak purity of the spiked analytes was used as an indication of specificity. 

#### 4.5.2. Calibration Model

Appropriate dynamic ranges for each analyte were considered. According to the spiking procedure all analytes were spiked at all levels e.g., folic acid was spiked from 2.5–50,000 ng/mL but the calibration model was evaluated from 5–2000 ng/mL, as this was the expected range in human plasma. Each calibration model was evaluated by linear or non-linear regression, using 1/y, none or 1/x weighting for minimizing the back calculated values (BCVs) but retaining R2 > 0.99. The acceptance criterion was %Er < 15% (<20% for the LLOQ).

#### 4.5.3. Recovery

Recovery was assessed at the LLOQ, ULOQ, and the appropriate QCs (as found in Supplementary S1). The recovery was calculated as the ratio of the corresponding signal of a pooled sample that had been pre-spiked and processed to the corresponding pooled sample that had been processed by the SPE method described and then spiked at the selected level. The basal signal was subtracted in both cases.

#### 4.5.4. Accuracy

Accuracy was assessed at the respective QC levels (n = 5), both inter day but also within five consecutive days (inter day). The results were expressed as relative percentage error (%Er). The acceptance criteria were set as %Er < 15% (and <20% at the LLOQ).

#### 4.5.5. Precision

The precision was assessed as repeatability (n = 5) and intermediate precision (n = 5), expressed by the %Relative Standard Deviation (%RSD). The acceptance criteria were <20% for the LLOQ and <15% in all other cases.

#### 4.5.6. Sensitivity

The required lower limits of quantification (LLOQ) for each analyte were targeted according to the expected literature values. The precision and accuracy were estimated at the LLOQ according to the regulatory bodies’ implementation. The LLOQ derived from the ratio of the response standard deviation to the slope of the corresponding calibration curve multiplied by five (LOD by 3.3). For the non-linear models, the slope was calculated from the linear part of the curve (as determined by linear regression analysis, considering the points that afforded a correlation coefficient >0.99). The instrumental sensitivity was evaluated in order to gain insight into the detection system capability for the current analysis using standard solutions (Supplementary S1) 

#### 4.5.7. Matrix Effect

The matrix effect was calculated based on the area ratio of samples (QC levels) that were spiked after the SPE processing of the pooled to the standard solution at the same levels.

#### 4.5.8. Stability

The stability was evaluated through freeze–thaw cycles, for the bench top conditions and the in-process stability. The stability of consecutive thaw-refrigeration cycles was carried out within 36 h at three concentration levels (QCL, QCM, and QCH with the QCM being the middle QC when more than one QC levels existed), where every 12 h the samples were thawed for 10 min and cooled again. The in-process stability examined the processed sample stability in the autosampler and was performed at three QC levels, per hour from the time they were prepared, i.e., for 12 h in total. 

#### 4.5.9. Dilution Integrity

The proper way to calculate the dilution integrity is demonstrated by spiking plasma samples with the analyte concentrations above their ULOQ (5-fold and 10-fold higher) and then diluting with blank plasma to levels that correspond to the calibration curve. In the present study due to the heterogeneity in the basal levels range of each metabolite, a wide concentration range for the calibration curve was employed (2.5–50,000 ng/mL) for all metabolites, i.e., all metabolites were spiked at the aforementioned range. Nevertheless, for the linearity assessment of each metabolite, only the levels corresponding to its respective dynamic range were considered. Thus, all metabolites were spiked at two high levels of 25,000–50,000 ng/mL to accommodate betaine, which exhibits very high basal plasma levels. The dilution integrity study of betaine was not performed as the 5-fold and 10-fold concentrations above the ULOQ would have been unrealistically high. The upper levels of the wide concentration range for all the analytes except betaine were considered as the dilution integrity levels and extended calibration curves including those levels were constructed to show the linearity of the response at levels higher than the ULOQ.

#### 4.5.10. Incurred Sample Reanalysis

All samples used for the assessment of the basal levels, i.e., the unknown samples, were reanalyzed on another laboratory day and the percentage difference between the two measurements was calculated. The exclusion criterion was set at <20% difference between two samples, with no more than 33% rejection rate for all samples. 

#### 4.5.11. Robustness

The robustness was evaluated causing deliberate ±10% changes at the concentration of the mobile phase, the desolvation temperature of the MS, and the mobile phase flow, since these parameters were found to be important during method optimization. The results were evaluated as %RSD, with exclusion criterion set <15%. 

#### 4.5.12. Analysis Timescale

The LC-MS/MS analysis run time was no larger than 10 min. As the sample pretreatment required less than half the time of the instrumental analysis (~5 min), nearly 150 samples can be analyzed over 24 h, which renders the methodology as high throughput.

## Figures and Tables

**Table 1:** Quality characteristics of the calibration curves of the substances. The actual dynamic range of the B12 calibration curve was far higher than the corresponding plasma levels of the substance, therefore it was not considered. * q = quadratic l = linear; ** SE = standard error; *** BCV’s = back calculated values.

Analyte	Type *	Weight	R^2^	S_y/x_ $\left(\times 10^{-5}\right)$	B_0_ ± SE ** $\left(\times 10^{-4}\right)$	B_1_ ± SE ** $\left(\times 10^{-5}\right)$	B_2_ ± SE ** $\left(\times 10^{-11}\right)$	Range (ng/mL)	BCVs ***
5-MeTHF	l	-	0.998	206.6	−27 ± 11	1.62 ± 0.03	-	50–7500	100%
Folic Acid	q	1/x	0.998	3.1	48 ± 82	1.02 ± 0.11	−80.5 ± 71.0	10–2000	80%
SAM	q	-	0.998	224.2	316 ± 9	0.26 ± 0.77	3.4 ± 0.3	10–50,000	75%
SAH	l	1/y	0.992	502.0	3103 ± 697	428 ± 160	-	10–50,000	85%
dTMP	q	1/y	0.992	374.1	2762 ± 1366	35.78 ± 2.25	−1.6 ± 1.5	2.5–50,000	80%
Phosphο	l	-	0.998	123.5	62 ± 43	0.17 ± 0.02	-	250–50,000	90%
DΜG	l	-	0.998	264.3	748 ± 88	0.00 ± 0.33	-	100–50,000	85%
Betaine	q	-	0.998	263.4	2311 ± 20	−0.01 ± 0.22	0.08 ± 0.42	2000–50,000	90%
B12	l	-	0.997	490.3	5 ± 3	0.00 ± 0.01	-	5000–50,000	0%

**Table 2:** Basal levels of analytes in the bloodstream of normal blood donors. The calculation was performed to gain insight into the levels of the analytes as assessed by the developed methodology compared to the corresponding ones determined in the literature.

Analyte	5-MeTHF	Folic Acid	SAM	SAH	PHOSPHO	BETAINE	DMG	dTMP
	(ng/mL)	(ng/mL)	(ng/mL)	(ng/mL)	(ng/mL)	(ng/mL)	(ng/mL)	(ng/mL)
Sample 1	164	18.3	75.8	38.0	335	1901	194	14.4
Sample 2	399	62.4	25.9	102	506	2666	105	7.6
Sample 3	255	21.8	75.8	56.2	438	4504	198	12.4
Sample 4	204	15.6	55.3	43.3	294	9067	169	8.2
Sample 5	199	81.5	87.0	32.0	421	3425	278	29.0
Sample 6	167	13.6	34.4	25.4	493	1921	576	34.5
STDEV	88.6	28.9	24.7	27.7	85.0	2709	167	11.3
AVG	231	35.5	59.0	49.4	415	3914	253	17.6
%RSD	38.2	81.6	41.9	56.1	20.5	69.2	65.9	64.0

**Table 3:** Mass spectrometry parameters used in the analysis.

Analyte	Precursor Ion (m/z)	Product Ion (m/z)	Dwell Time (s)	Cone Voltage (V)	Fragmentation Energy (V)
N,N-dimethylglycine	104.0	58.0	0.022	46	12
betaine	118.0	58.9	0.022	28	16
phosphocholine	184.0	86.0	0.022	34	16
dTMP	323.0	80.9	0.022	24	16
S-adenosylmethionine	385.1	88.0	0.070	34	44
S-adenosylhomocysteine	399.1	250.1	0.070	36	14
folic acid	442.1	295.1	0.022	28	12
5-methyltetrahydrofolic acid	460.1	313.1	0.022	34	20
B12 (cyanocobalamin)	678.6	147.1	0.022	46	42
daptomycin	460.1	313.1	0.022	28	16

## Data Availability

The data presented in this study are available in this article and supplementary material.

## Supplementary Materials

The following are available online at https://www.mdpi.com/article/10.3390/metabo11070416/s1, Table S1: Recovery and the corresponding uncertainty of the analytes, Table S2: Accuracy of the analysis expressed as inter- and intra-day% Relative Error, Table S3: Precision data (repeatability and intermediate precision) expressed as RSD (%), Table S4: Instrumental and method sensitivity described by the LOQ, Table S5: Matrix effect of the signal expressed as % signal recovery along with the corresponding % RSD, Table S6a: Freeze-thaw stability data, Table S6b: Bench top stability data, Table S7: Robustness data on changing desolvation temperature, flow rate, and buffer concentration deliberately. The results are expressed as %RSD. Table S8: Linear approximation of calibration curves in order to achieve the base levels of the circulating analytes in blood.
