# Supplementary material for: Targeted Metabolomics: The LC-MS/MS Based Quantification of the Metabolites Involved in the Methylation Biochemical Pathways
Source: Metabolites. 2021 Jun 24;11(7):416. doi: 10.3390/metabo11070416 (PMC8307054; doi:10.3390/metabo11070416)
Supplement: Supplementary file 1 [file metabolites-11-00416-s001.zip › metabolites-1239080-supplementary.pdf]

Contents

Supplementary S1 2

Table S1. Recovery and the corresponding uncertainty of the analytes 3

Table S2. Accuracy of the analysis expressed as inter- and intra- day % Relative Error 6

Table S3. Precision data (repeatability and intermediate precision) expressed as RSD (%) 10

Table S4. Instrumental and method sensitivity described by the LOQ 15

Table S5. Matrix effect of the signal expressed as % signal recovery along with the corresponding % RSD 16

Table S6a. Freeze-thaw stability data 19

Table S6b. Bench top stability data 20

Table S7. Robustness data changing desolvation temperature, flow rate and buffer concentration deliberately. The results are expressed as %RSD. 23

Table S8. Linear approximation of calibration curves in order to achieve the base levels of the circulating analytes in blood 24

Supplementary S1

The calibrators were diluted in water at the following levels:

2.5, 5, 10, 25, 50, 75, 100, 250, 500, 750, 1000, 1500, 2000, 5000, 7500, 10000, 20000, 30000, 35000, 40000, 45000, 50000 ng/mL containing the IS at the 20 µg/mL level.

Due to the fact that the analytes are present in hugely different levels in plasma, the use of such a large number of calibrators has been deemed as necessary.

Table S1. Recovery and the corresponding uncertainty of the analytes

**5-MeTHF**	**C(ng/ml)**	**% Recovery ± STD**
$LLOQ_1$	15	57.4 ±11.60
QCL_1	80	58.16±8.93
QCL_2	800	65.67±4.68
QCM	1800	71.07±3.16
QCH	3000	71.93±5.50
ULOQ	6000	80.49±7.83

**SAH**	**C(ng/ml)**	**% Recovery ± STD**
$LLOQ_1$	15	62.53±13.20
QCL_1	80	63.09±9.99
QCL_2	800	48.32±9.07
QCM_1	1800	65.42±7.18
QCM_2	3000	73.11±4.07
QCM_3	6000	56.04±6.63
QCH_1	9000	70.13±8.97
QCH_2	25000	70.54±3.27
ULOQ	45000	74.79±5.52

SAM	C(ng/ml)	% Recovery ± STD
$LLOQ_1$	15	64.20±13.63
QCL_1	80	56.22±11.90
QCL_2	800	80.27±4.49
QCM_1	1800	79.81±3.57
QCM_2	3000	81.74±9.46
QCM_3	6000	81.96±8.35
QCH_1	9000	82.07±7.24
QCH_2	25000	76.43±6.13
ULOQ	45000	91.08±5.09

Phosphocholine	C(ng/ml)	% Recovery ± STD
LLOQ	800	57.36±10.43
QCL	1800	53.35±11.90
QCM_1	3000	70.49±7.69
QCM_2	6000	68.88±1.51
QCH_1	9000	69.78±7.54
QCH_2	25000	78.66±2.56
ULOQ	45000	91.30±5.58

Betaine	C(ng/ml)	% Recovery ± STD
LLOQ	3000	90.51±5.25

QCL	6000	84.46±8.92
QCM	9000	82.26±7.93
QCH	25000	92.49±2.59
ULOQ	45000	102.34±2.41

**Dimethylglycine**	**C(ng/ml)**	**% Recovery ± STD**
LLOQ	800	70.25±9.25
QCL	1800	67.59±2.86
QCM_1	3000	51.38±7.08
QCM_2	6000	69.48±2.35
QCH_1	9000	74.09±7.95
QCH_2	25000	68.53±8.25
ULOQ	45000	63.86±3.55

Table S2. Accuracy of the analysis expressed as inter- and intra- day % Relative Error

**5-MeTHF**	**C(ng/ml)**	**Error (%) Intra day**	**Error (%) Inter day**
LLOQ	80	4.73	6.07
QCL	800	4.87	-4.56
QCM	1800	-1.56	7.98
QCH	3000	2.09	3.41
ULOQ	6000	3.41	9.03

**Folic Acid**	**C(ng/ml)**	**Error (%) Intra day**	**Error (%) Inter day**
LLOQ$_1$	15	-4.66	-8.14
QCM	80	4.25	5.47
QCH	800	2.89	-6.23
ULOQ	1800	-7.44	8.86

**SAM**	**C(ng/ml)**	**Error (%) Intra day**	**Error (%) Inter day**
LLOQ$_1$	15	8.42	-9.09
QCL$_1$	80	-2.29	-8.41

QCL_2	800	-4.77	-6.85
QCM_1	1800	-2.61	-5.29
QCM_2	3000	3.25	7.52
QCM_3	6000	4.19	9.38
QCH_1	9000	-3.17	8.20
QCH_2	25000	6.42	-3.98
ULOQ	45000	7.51	-8.09

**SAH**	**C(ng/ml)**	**Error (%) Intra day**	**Error (%) Inter day**
$LLOQ_1$	15	-7.58	-8.14
QCL_1	80	-2.36	-6.41
QCL_2	800	-4.69	-9.07
QCM_1	1800	-2.91	-3.32
QCM_2	3000	6.48	-2.98
QCM_3	6000	3.88	3.61
QCH_1	9000	-3.87	2.79
QCH_2	25000	2.65	-2.09
ULOQ	45000	-5.68	4.35

**dTMP**	**C(ng/ml)**	**Error (%) Intra day**	**Error (%) Inter day**

$LLOQ_1$	15	-8.59	10.47
QCL_1	80	4.26	-5.91
QCL_2	800	2.78	9.08
QCM_1	1800	5.21	-7.15
QCM_2	3000	-5.08	-3.02
QCM_3	6000	-2.08	7.80
QCH_1	9000	2.93	7.95
QCH_2	25000	7.22	5.81
ULOQ	45000	-3.59	-8.68

**Phosphocholine**	**C(ng/ml)**	**Error (%) Intra day**	**Error (%) Inter day**
LLOQ	800	4.22	7.29
QCL	1800	3.08	-8.92
QCM_1	3000	-4.71	9.22
QCM_2	6000	6.60	9.33
QCH_1	9000	6.79	-8.31
QCH_2	25000	4.08	8.11
ULOQ	45000	6.29	8.85

**Dimethylglycine**	**C(ng/ml)**	**Error (%) Intra day**	**Error (%) Inter day**

LLOQ	800	-8.69	-6.46
QCL	1800	-5.22	-12.41
QCM$_1$	3000	-6.77	6.98
QCM$_2$	6000	2.81	7.41
QCH$_1$	9000	-5.33	9.84
QCH$_2$	25000	-7.84	11.13
ULOQ	45000	-8.69	10.72

**Betaine**	**C(ng/ml)**	**Error (%) Intra day**	**Error (%) Inter day**
LLOQ	3000	-3.54	-5.26
QCL	6000	-2.19	4.08
QCM	9000	4.13	5.29
QCH	25000	-3.85	-7.49
ULOQ	45000	-5.54	-8.22

Table S3. Precision data (repeatability and intermediate precision) expressed as RSD (%)

**5-MeTHF**	**C(ng/ml)**	**RSD (%) Repeatability**	**RSD (%) Intermediate precision**
LLOQ	80	1.72	6.97
QCL2	800	3.16	7.33
QCM	1800	2.17	7.40
QCH	3000	2.87	5.40
ULOQ	6000	6.76	6.81

**Folic Acid**	**C(ng/ml)**	**RSD (%) Repeatability**	**RSD (%) Intermediate precision**
LLOQ1	15	3.66	7.54
QCM	80	1.43	5.97
QCH	800	4.74	6.79
ULOQ	1800	1.71	6.38

SAM	C(ng/ml)	RSD (%) Repeatability	RSD (%) Intermediate precision
$LLOQ_1$	15	4.82	13.41
QCL_1	80	7.41	10.42
QCL_2	800	9.61	11.91
QCM_1	1800	1.95	3.87
QCM_2	3000	9.71	10.58
QCM_3	6000	5.05	9.08
QCH_1	9000	8.51	9.69
QCH_2	25000	4.30	6.71
ULOQ	45000	7.36	8.74

SAH	C(ng/ml)	RSD (%) Repeatability	RSD (%) Intermediate precision
$LLOQ_1$	15	8.85	6.99
QCL_1	80	6.12	7.11
QCL_2	800	5.27	6.98
QCM_1	1800	11.8	16.10
QCM_2	3000	3.19	8. 25
QCM_3	6000	2.07	9.32

QCH$_1$	9000	6.47	8.16
QCH$_2$	25000	8.55	8.37
ULOQ	45000	5.73	7.41

**Betaine**	**C(ng/ml)**	**RSD (%) Repeatability**	**RSD (%) Intermediate precision**
LLOQ	3000	4.04	7.72
QCL	6000	1.50	6.97
QCM	9000	5.98	7.67
QCH	25000	1.71	8.16
ULOQ	45000	2.83	6.03

**dTMP**	**C(ng/ml)**	**RSD (%) Repeatability**	**RSD (%) Intermediate precision**
LLOQ$_1$	15	7.15	9.54
QCL$_1$	80	4.19	5.55
QCL$_2$	800	3.09	6.48
QCM$_1$	1800	4.34	6.72
QCM$_2$	3000	9.77	8.06

QCM_3	6000	3.77	7.49
QCH_1	9000	9.90	3.68
QCH_2	25000	4.87	8.15
ULOQ	45000	8.01	9.54

**Phosphocholine**	**C(ng/ml)**	**RSD (%) Repeatability**	**RSD (%) Intermediate precision**
LLOQ	800	8.11	13.51
QCL	1800	7.43	6.15
QCM_1	3000	2.26	5. 37
QCM_2	6000	4.02	9.32
QCH_1	9000	7.31	8.43
QCH_2	25000	7.27	11.72
ULOQ	45000	4.20	8. 13

**Dimethylglycine**	**C(ng/ml)**	**RSD (%) Repeatability**	**RSD (%) Intermediate precision**
LLOQ	800	2.17	9.09
QCL	1800	6.54	10.68
QCM_1	3000	0.51	3.03

QCM_2	6000	3.07	6.83
QCH_1	9000	7.33	11.82
QCH_2	25000	4.75	6.18
ULOQ	45000	6.15	8.57

Table S4. Instrumental and method sensitivity described by the LOQ.

**Analyte**	**Instrumental LOQ (ng/ml)**	**Methodological LOQ (ng/ml)**
5-MeTHF	25	80
Folic Acid	5	15
SAM	5	15
SAH	5	15
d-TMP	10	15
Phosphocholine	250	800
Dimethylglucine	50	150
Betaine	5	3000

Table S5. Matrix effect of the signal expressed as % signal recovery along with the corresponding % RSD

**5-MeTHF**	**C(ng/ml)**	**% Recovery ± STD**
LLOQ	15	100.19±12.91
QCM	1800	91.25± 4.78
QCH	3000	97.65±3.42

**Folic Acid**	**C(ng/ml)**	**% Recovery ± STD**
LLOQ	15	82.87±13.24
QCM	1800	109.55±5.25
QCH	3000	87.63±3.95

**SAM**	**C(ng/ml)**	**% Recovery ± STD**
LLOQ	15	90.41±13.82
QCM	3000	74.22±5.96
QCH	25000	65.88±6.23

**SAH**	**C(ng/ml)**	**% Recovery ± STD**

LLOQ	15	109.04±15.02
QCM	3000	82.20±8.77
QCH	25000	93.19±3.37

**Phosphocholine**	**C(ng/ml)**	**% Recovery ± STD**
LLOQ	15	107.62±13.67
QCM	3000	105.93±7.98
QCH	25000	85.38±2.42

**dTMP**	**C(ng/ml)**	**% Recovery ± STD**
LLOQ	15	107.98± 14.72
QCM	3000	109.36± 2.02
QCH	25000	102.61±3.34

**Dimethylglucine**	**C(ng/ml)**	**% Recovery ±STD**
LLOQ	15	104.04±7.32
QCM	3000	125.71±2.52
QCH	25000	114.04±2.05

**Betaine**	**C(ng/ml)**	**% Recovery ± STD**
LLOQ	3000	103.81±14.38
QCM	9000	109.82±9.12
QCH	25000	111.29±5.28

Table S6a. Freeze-thaw stability data

Betaine	C(ng/ml)	% RSD
LLOQ	3000	17.65
QCM	9000	22.85
QCH	25000	37.49

Dimethylglycine	C(ng/ml)	% RSD
LLOQ	15	24.14
QCM	3000	52.70
QCH	25000	24.05

Phosphocholine	C(ng/ml)	% RSD
LLOQ	15	34.29
QCM	3000	32.97
QCH	25000	50.62

SAM	C(ng/ml)	% RSD
LLOQ	15	15.53
QCM	3000	12.12
QCH	25000	16.97

SAH	C(ng/ml)	% RSD

LLOQ	15	35.55
QCM	3000	33.17
QCH	25000	20.09

**dTMP**	**C(ng/ml)**	**% RSD**
LLOQ	15	61.45
QCM	3000	62.94
QCH	25000	50.82

**5MeTHF**	**C(ng/ml)**	**% RSD**
LLOQ	15	49.07
QCM	3000	29.68
QCH	25000	36.74

**Folic Acid**	**C(ng/ml)**	**% RSD**
LLOQ	15	17.58
QCM	3000	41.18
QCH	25000	20.12

Table S6b. Bench top stability data

**Betaine**	**C(ng/ml)**	**% RSD**
LLOQ	3000	11.84
QCM	9000	1.27
QCH	25000	3.89

**Dimethylglycine**	**C(ng/ml)**	**% RSD**
LLOQ	15	9.81
QCM	3000	4.14
QCH	25000	3.31

**Phosphocholine**	**C(ng/ml)**	**% RSD**
LLOQ	15	12.23
QCM	3000	6.89
QCH	25000	7.61

**SAM**	**C(ng/ml)**	**% RSD**
LLOQ	15	7.55
QCM	3000	4.96
QCH	25000	4.93

**SAH**	**C(ng/ml)**	**% RSD**

LLOQ	15	8.39
QCM	3000	2.38
QCH	25000	1.83

**dTMP**	**C(ng/ml)**	**% RSD**
LLOQ	15	3.94
QCM	3000	4.55
QCH	25000	10.77

**5MeTHF**	**C(ng/ml)**	**% RSD**
LLOQ	15	3.99
QCM	3000	5.17
QCH	25000	1.72

**Folic Acid**	**C(ng/ml)**	**% RSD**
LLOQ	15	12.02
QCM	3000	9.53
QCH	25000	6.54

Table S7. Robustness data changing desolvation temperature, flow rate and buffer concentration deliberately. The results are expressed as %RSD.

	**Desolvation Temperature**	**Flow rate**	**Buffer concentration**
**Analyte**	**% RSD**		
5-MeTHF	1.78	6.96	2.93
Folic Acid	10.16	7.55	4.82
SAM	9.34	6.54	2.37
SAH	8.81	8.00	14.49
d-TMP	13.81	7.48	15.20
Phosphocholine	11.47	11.07	24.59
Dimethylglycine	3.27	11.98	13.32
Betaine	0.67	6.86	17.47

Table S8. Linear approximation of calibration curves in order to achieve the base levels of the circulating analytes in blood

Analyte	R^2	$S_{y/x}$	B_0	B_1	Range (ng/ml)
5-MeTHF	0.998	0.0020	(0.0022±0.0009)	(1.616 ± 0.029) 10^{-5}	50-7500
Folic Acid	0.998	0.0007	(0.283± 0.2977)10^{-3}	(87.58±3.46) 10^{-7}	10-2000
SAM	0.998	0.0072	(0.0033±0.0022)	(3.455±0.116) 10^{-6}	10-30000
SAH	0.992	0.0287	(0.103±0.0697)10^{-5}	(305.71±2.92)10^{-7}	10-20000
dTMP	0.997	0.0001	(6.407 ± 4.063)10^{-5}	(328.77 ± 5.13)10^{-8}	2.5-3000
Phosphocholine	0.995	0.0022	(9.637 10^{-4} ± 3.722 $10^{-8)}$	(1.765±0.024) 10^{-6}	250-50000

DMG	0.998	0.0200	(0.0082 ±0.0007)	$(3.553 \pm 0.105)\ 10^{-6}$	100-50000
Betaine	0.997	0.0193	(0.0234 ± 0.0162)	$(7.655 \pm 0.462)\ 10^{-6}$	2000-50000
